# Comparison of the effectiveness of sodium-tri-metaphosphate-treated varnish containing eggshell and membrane powder and bioactıve glass varnish with fluoride varnish in preventing erosion: *in vitro*

**DOI:** 10.1007/s10266-025-01065-w

**Published:** 2025-03-01

**Authors:** Hande Yalçınkaya Cengiz, Hayriye Esra Ülker, Ercan Durmuş, İlhami Çelik

**Affiliations:** 1 Faculty of Dentistry Department of Restorative Dentistry, Izmir Katip Celebi University, İzmir, Turkey; 2 Faculty of Dentistry Department of Restorative Dentistry, Selcuk University, Konya, Turkey; 3 Faculty of Dentistry Department of Oral and Maxillofacial Surgery, Selcuk University, Konya, Turkey; 4 Faculty of Veterinary Department of Histology and Embryology, Selcuk University, Konya, Turkey

**Keywords:** Erosion, pH, Remineralization, Demineralization, Fluoride, Bioactive varnish

## Abstract

The aim of this in vitro study was to compare the efficacy of sodium-tri-metaphosphate-treated varnish containing eggshell and membrane powder and bioactive glass varnish with fluoride varnish in preventing erosion. Two windows were created on the buccal surface of 72 molars for the erosion cycle. One of the windows was treated with fluoride varnish [(FV, Metroberry, Imicryl), varnish containing STMP-treated eggshell and membrane powder (EPV, BioViera, Imicryl) and bioactive glass varnish (BAG, Polimo, Imicryl)] while the other window was used as control. Acidic syrup (Atarax) was applied 3 times a day and acidic drink (Coca Cola) 4 times a day for 5 days. The samples were analyzed by SEM (*n* = 1), ATR-FTIR (*n* = 6) and Vicker’s hardness (*n* = 5). Data were analyzed by one-way ANOVA and post hoc Tukey test. The protective effects of the varnishes were observed in the SEM images obtained. There was a difference between the FV and EPV groups in the 875 cm^−1^ v_2_ CO_3_^−2^ peak spectrum and microhardness values (*p* < 0.05). There was a difference between FV and FV-C in the carbonate v_2_ band in the acidic beverage demineralised varnish groups (*p* < 0.05). Other band areas and CO_3_^−2^ /PO_4_^−3^ ratios of the varnish areas against the erosion and control areas showed similar results (*p* > 0.05). Microhardness analyses showed that the BAG group demineralized with acidic syrup and the FV group demineralized with acidic drink were similar to the control group (*p* > 0.05). The varnishes tested in our study showed a similar protective effect against erosion as fluoride varnishes.

## Introduction

Tooth erosion is a multifactorial condition, defined as the loss of tooth hard tissues by chemical processes that do not involve bacteria [1, 2]. It is of the utmost importance to identify potential risk factors in order to implement preventative measures. The rise in consumption of acidic foods and beverages in recent years, coupled with changes in lifestyle and circumstances, has led to an increase in the prevalence of erosion [3–6]. A number of studies have been conducted with the objective of developing methods to prevent dental erosion and to strengthen teeth against this process [7, 8]. The discovery that the mechanism of hard tissue destruction is similar to that of initial caries lesions has prompted the exploration of remineralization techniques for the treatment of these lesions, with the development of materials designed for non-invasive applications [7].

Given the irreversible nature of tooth erosion, it is of the utmost importance to implement preventative treatments. Fluoride, a halogen element, is the most crucial active ingredient in protective dental applications. It is understood to reinforce tooth structure against acid attack by reducing the permeability of enamel [9, 10]. The principal applications of fluoride are in the form of solutions, gels, varnishes, fluoride toothpastes and mouthwashes.

**Table 1:** List of Groups and Abbreviations Used in the Study

Abbreviations	Groups
FV	Fluoride varnish group
BAG	Bioactive glass varnish group
EPV	STMP treated egg shell and membrane powder varnish group

Fluoride has been identified as a crucial element in the process of remineralization, and has been employed to facilitate the formation of fluorapatite, which enhances the acid resistance capacity of tooth enamel [11]. Fluoride has been demonstrated to inhibit mineral loss from the enamel surface. This occurs via two principal mechanisms: first, by inducing surface adsorption on demineralised crystals and attracting ions from the oral fluid; and second, by forming fluorapatite on the enamel surface, thus increasing the enamel’s resistance to acid attack [12, 13]. Fluoride has been demonstrated to exert a limited effect on apatite dissolution rather than facilitating mineralization to offset the loss of minerals within the apatite crystal [14]. Additionally, it was determined that the treatment had a notable impact on the smooth surface of the enamel though its efficacy was comparatively diminished in the context of grooves and pits [15]. The initial high rates of fluorapatite deposition in the surface layer may impede the diffusion of ions into the underlying lesion, which could result in incomplete remineralization [16]. Furthermore, the ingestion of fluoride during the developmental phase of the dentition elevates the probability of developing dental fluorosis [16, 17]. Consequently, research is being conducted on alternative agents that can enhance remineralization without the attendant risks associated with fluoride [18].

A bioactive glass (45S5) has been developed for use in dentistry and has been the subject of numerous studies aimed at demonstrating its efficacy in remineralizing initial lesions [19–21]. The glass has demonstrated the potential to induce apatite formation when in contact with saliva or other physiological fluids. The formation of apatite occurs in two forms: hydroxyapatite and fluorapatite. The latter is contingent upon the inclusion of fluoride in the chemical composition of the glass structure [22]. Fluoride-containing glasses have been demonstrated to possess ‘smart’ properties, exhibiting increased remineralization activity in low pH environments ^22^. Consequently, it has been incorporated into a range of products, including toothpastes, prophylactic gels and dental materials, for the treatment of enamel demineralization [23].

An additional option is eggshell powder (EP), which is a rich source of calcium, phosphorus, strontium, zinc, fluoride and copper. These elements can facilitate remineralization [24, 25]. It has been demonstrated that this substance increases bone density [26], reduces resorption, and alleviates pain. This substance has been demonstrated to possess an antirachitic effect in both animal (mice) and human models [27]. The potential applications of EPs in dentistry have been the subject of investigation, with studies examining their use as a pulp coating material [28, 29], as a protective varnish in enamel erosion [18], and as bone grafts [30]. The majority of research into eggshells has focused on their remineralization abilities. It has recently been reported that eggshells contain a substantial calcium content, which can be harnessed as a rich biological structure to facilitate the remineralization of caries lesions [31].

Another compound with potential for use in remineralization is sodium trimetaphosphate (STMP), which has the ability to bind to the enamel surface and remain adsorbed for longer periods of time. Another compound with potential for use in remineralization is sodium trimetaphosphate (STMP), which has the ability to bind to the enamel surface and remain adsorbed for longer. STMP (Na_3_P_3_O_9_) binds strongly to the phosphate on the enamel (which inhibits the release of calcium and phosphate from the crystal) and leads to the formation of a layer on the enamel surface that limits the diffusion of acid ions.This results in the formation of a protective layer that limits the diffusion of ions during a cariogenic loading process. This protective barrier serves to impede the diffusion of calcium and fluoride ions from the enamel [32].

Various measurement methods used in the evaluation of erosion on tooth surfaces include Surface Microhardness Measurement, Scanning Electron Microscopy (SEM), and Attenuated Total Reflectance Fourier-Transform Infrared Spectroscopy (ATR-FTIR) [33]. Surface microhardness measurement is used to determine the loss of hardness in the enamel and the hardness of the tooth is evaluated by measuring the penetration depth. This method is easy to apply and low cost, but has limitations such as the fact that it can only be measured to a depth of 25 μm and cannot give precise results on dentin [34]. SEM allows the topographic structure of the tooth surface to be examined with high resolution and allows detailed observation of erosion and morphological changes in tooth enamel. However, it cannot be used in in vivo studies because the samples must be coated with metal [35]. ATR-FTIR, on the other hand, analyzes the chemical components in dental materials and provides fast and precise results without the need for sample preparation. Thus, it can be used to evaluate the chemical changes of tooth erosion [36]. These methods allow the study of different aspects of erosion on the tooth surface and are often used in combination to make more comprehensive assessments [37].

The objective of this in vitro study was to assess the efficacy of fluoride varnish, bioactive glass varnish, sodium-tri-metaphosphate-treated varnishes and varnishes containing eggshell and membrane powder in protecting against erosion caused by soft drinks and acidic drugs. This was achieved through the use of scanning electron microscopy (SEM) images, attenuated total reflection Fourier-transform infrared spectroscopy (ATR-FTIR), and surface microhardness measurements.

The null hypothesis of this in vitro study is that there is no difference between the effects of fluoride varnish, bioactive glass varnish, sodium-tri-metaphosphate-treated varnishes, and varnishes containing eggshell and the membrane powder in terms of their ability to protect against erosion caused by non-alcoholic acidic beverages and acidic drugs.

## Materials and methods

Ethical approval for the study was granted by the Non-interventional Research Ethics Committee of Selçuk University Faculty of Dentistry (2021/25).

**Fig. 1:**
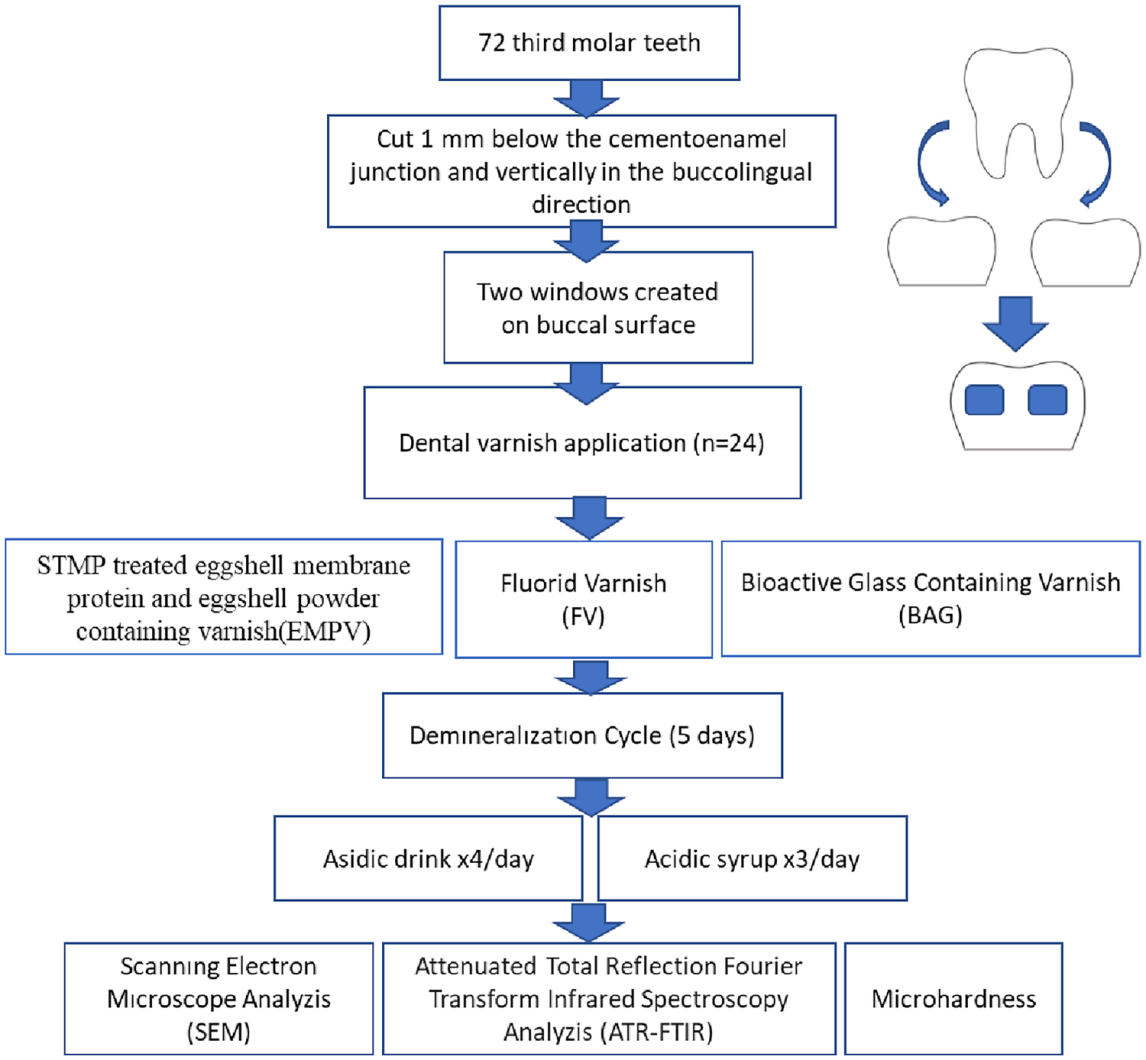
Schematic of Experimental Design

The process steps followed in this article are summarized in Fig. 1. Table 1

### Preparation of Enamel Samples

In this study, 72 molars with indications for extraction were used and the teeth were examined under a stereo microscope and those with fractures, cracks, caries, and restorations on the surface were excluded. The teeth were required to have morphologically appropriate buccal enamel surfaces with similar dimensions of 2.5 cm length, 1.2 cm width, and 1.0 cm thickness. The teeth were stored in 0.1% thymol solution at + 4ºC for a maximum of 2 months [38]. Tissue debris was removed with a periodontal curette, polishing rubber and pumice, and ground under water with 800–4000 grit silicon carbide sandpaper. The roots were cut with a water-cooled low-speed diamond saw and two enamel blocks of 8 mm × 8 mm × 4 mm were obtained from each tooth. Two coats of nail polish were applied to the enamel surfaces to prevent acid ingress into the dentinal tubules, and then a 2 × 3 mm window was cut in the buccal enamel. The samples were incubated in an oven at 37 °C.

### Application of varnish

Constituents, manufacturers, and batch numbers of the varnishes employed in the study are provided in Table 2. Prior to the application of the varnish, the samples were rinsed thoroughly with deionized water and randomly divided into three groups. The varnishes, which contained fluoride, bioactive glass, and STMP-treated eggshell and membrane powder, were applied to the right window of the samples for four minutes with the assistance of a microbrush. The other window served as the control group. Subsequently, all samples were incubated in artificial saliva at 37 °C for a period of 24 h. During the study phases, the formula of artificial saliva with a pH of 6.8 used during the study phases was 125.6 mg/L NaCl, 963.9 mg/L KCl, 227.8 mg/L CaCl_2_.2H_2_O, 178 mg/L NH_4_Cl, 189.2 mg/L KSCN, 336.5 mg/L Na_2_SO_4_, 200 mg/L, urea (CH4N2O), 630.8 mg/L NaHCO_3_, and 654.5 mg/L KH_2_PO_4_ [39].

### Demineralization and remineralization cycles

After dividing the teeth into three groups according to different varnish types, they were randomly divided into two subgroups, acidic beverage and syrup, and subjected to erosive cycles.

#### Acidic syrup cycle

The erosion protocol was adapted from previous studies [40–42] and syrup (pH 2.25) was applied by immersion in 3 ml of syrup for 1 min, 3 times a day for 5 days. The titratable acidity of the syrup was measured as 0.54 ± 0.01 ml with a volume of 0.1 mol NaOH to bring the pH to 7. The samples were washed with distilled water after each immersion and stored in artificial saliva at 37 °C.

#### Acidic drink cycle

Coca Cola drink (pH 2.6) was applied for 20 min by immersing the samples in 20 ml of the drink for 5 min, 4 times a day. The titratable acidity was measured as 3.3 ml with a volume of 0.1 mol NaOH to bring the pH to 7. The samples were washed with distilled water after each cycle and stored in artificial saliva at 37 °C. Samples were kept in airtight containers during erosion.

**Table 2:** Demineralization and remineralization agents used in the study, their contents, manufacturers, and lot numbers

Demineralization and remineralization materials	Contents	Manufacturer/lot number
Coca Cola^®^	Water, Sugar, Carbon dioxide, Coloring (Caramel), Acidity Regulator (Phosphoric Acid), Natural Flavorings, Caffeine (Max. 0,150 G/L)	Coca Cola Company, ABD/11201
Atarax^®^	Hydroxyzine Dihydrochloride, Ethanol, Sucrose, Sodium Benzoate, Levomenthol, Hazelnut Flavor and Purified Water	UCB PHARMA(İstanbul, Turkey)/26300
Bio Viera Varnish^®^	Organic Resin, Ethyl Alcohol, Xylitol, Flavor, Sodium TriMetaphosphate-treated eggshell membrane protein and Eggshell Powder	IMICRYL(Konya,Turkey)19,116
Metroberry Varnish^®^	Sodium fluoride 22,600 ppm, Solvent, Organic Resin, Xylitol, Flavoring	IMICRYL (Konya, Turkey)/18222
Poimo Varnish^®^	Sodium Fluoride, Tricalcium phosphate, Bioactive Glass, Ethyl Alcohol, Organic binder, Xylitol, Flavoring	IMICRYL (Konya, Turkey)/19043

### SEM analyses

In order to examine the surface properties of the enamel samples after a five-day remineralization –demineralization cycle, one tooth was washed with distilled water and the surfaces were cleaned with a soft cloth. The samples were then maintained in artificial saliva for a period of two days, after which they were rinsed with distilled water and placed in an incubator for a further two days to facilitate drying. The treated surface of the samples was coated with a gold plating device (Sputter Coater 108 Auto, Cressington Scientific Instruments Ltd, Watford, UK). Scanning electron microscopy (SEM) images were obtained at 5000 × and 10,000 × magnification using an Evo LS10 instrument (Carl Zeiss, Oberkochen, Germany).

### Attenuated total reflectance fourier-transform ınfrared spectroscopy (ATR-FTIR)

The alterations in the chemical configuration of the enamel were quantified through the utilization of an FTIR (Fourier-transform infrared spectroscopy-Spectrum 100, PerkinElmer Life and Analytical Sciences, USA) spectrophotometer, which was equipped with an ATR (Attenuated Total Reflection) unit (Pike Tech).

### Microhardness analyses

To assess the rate of enamel demineralization in the samples, the changes in microhardness were examined and the measurements were expressed as Vickers microhardness values. Microhardness measurements were performed using a microhardness analyzer with a Vickers tip (Hardway-mhvs 1000 AD, China). Measurements were made by applying a force of 100 g for 10 s. The first microhardness measurement was performed on intact enamel immediately after preparation of the specimens. The surfaces were cleaned after the acid drink and the erosion cycle. The microhardness values of the right and left windows were measured. 3 measurements were taken. Each pyramidal diamond indentation on the surface was measured at 40 × magnification.

### Statistical analysis

All statistical analyses were conducted using IBM SPSS Statistics, version 29.0.0 (IBM SPSS, Chicago, Illinois, USA). The normality of the distribution was evaluated through the implementation of Kolmogorov–Smirnov and Shapiro–Wilk tests, with a significance level of 5% (*α* = 0.05) established for all analytical procedures. The data obtained from the microhardness and ATR-FTIR measurements were found to be normally distributed. Subsequently, the differences in the results were determined by one-way analysis of variance (ANOVA). A post- hoc Tukey test was employed to ascertain the discrepancies between the various groups. Intragroup comparisons were made according to the ATR-FTIR results using a t test.

## Results

### SEM Results

**Fig. 2:**
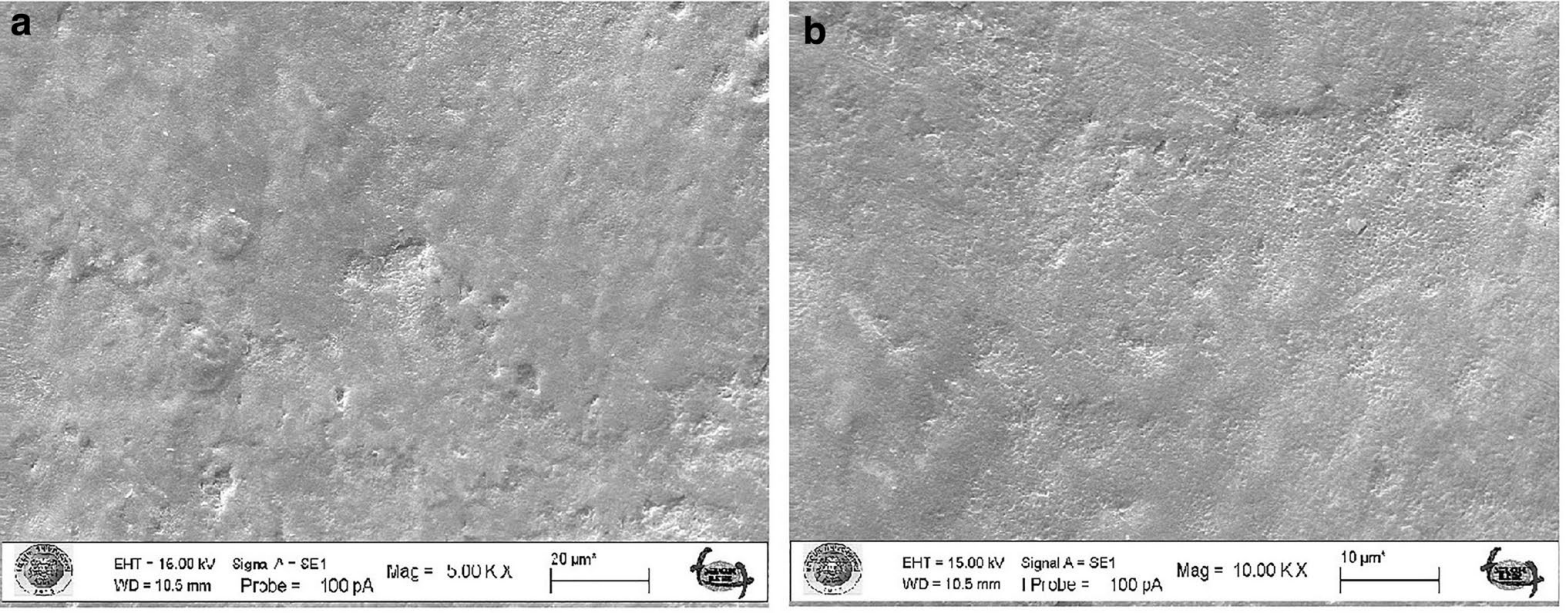
SEM image of intact enamel surface (× 5000) **b**. SEM image of intact enamel surface (× 10,000)

**Fig. 3:**
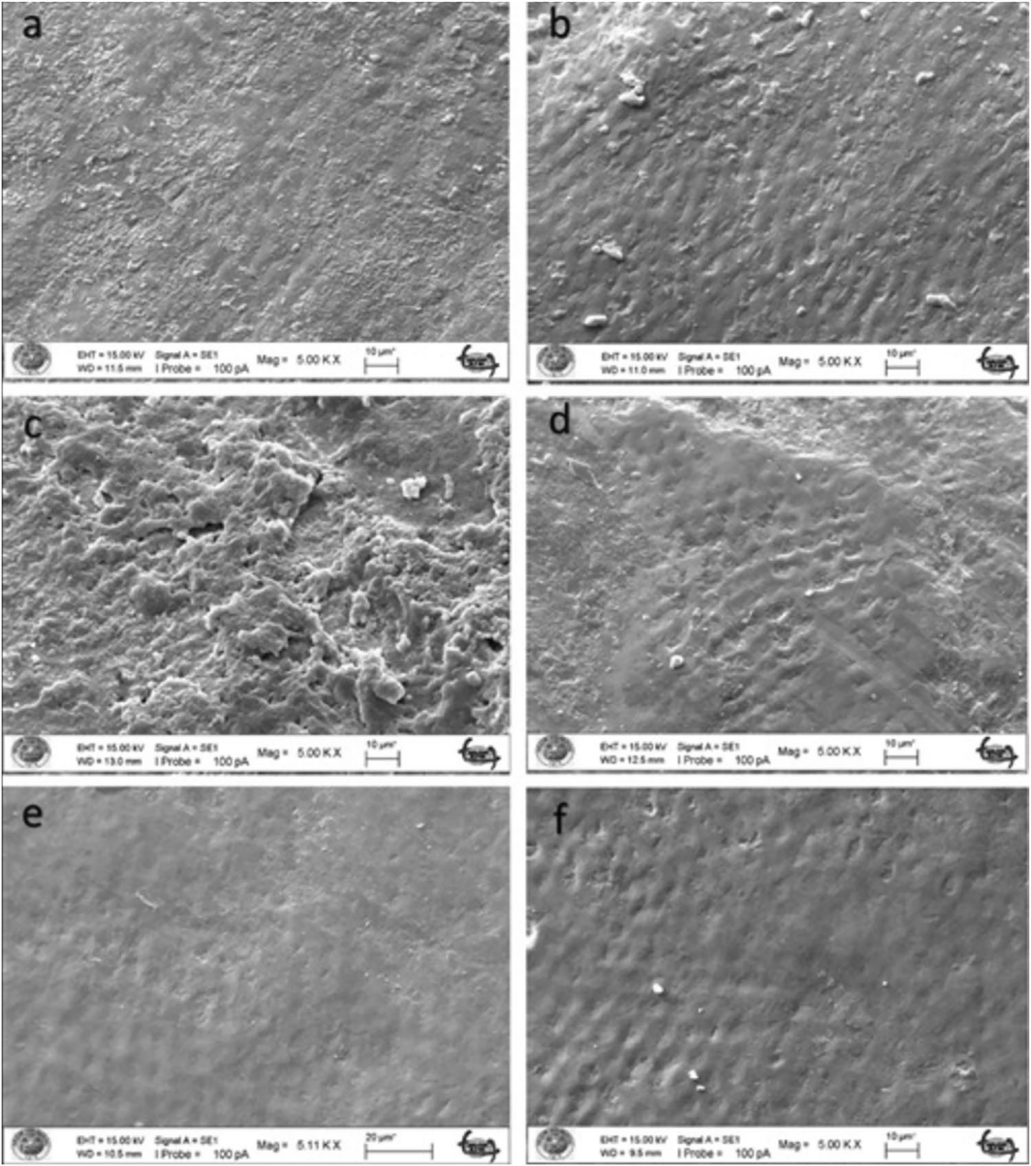
SEM image of enamel demineralised with acidic syrup. **a** FV applied enamel (× 5000). **b** Enamel without FV applied (× 5000). **c** SEM image of enamel with BAG varnish applied (× 5000). **d** Enamel without BAG applied (× 5000). **e** SEM image of enamel with EPV applied (× 5000). **f** Enamel without EPV applied (× 5000)

**Fig. 4:**
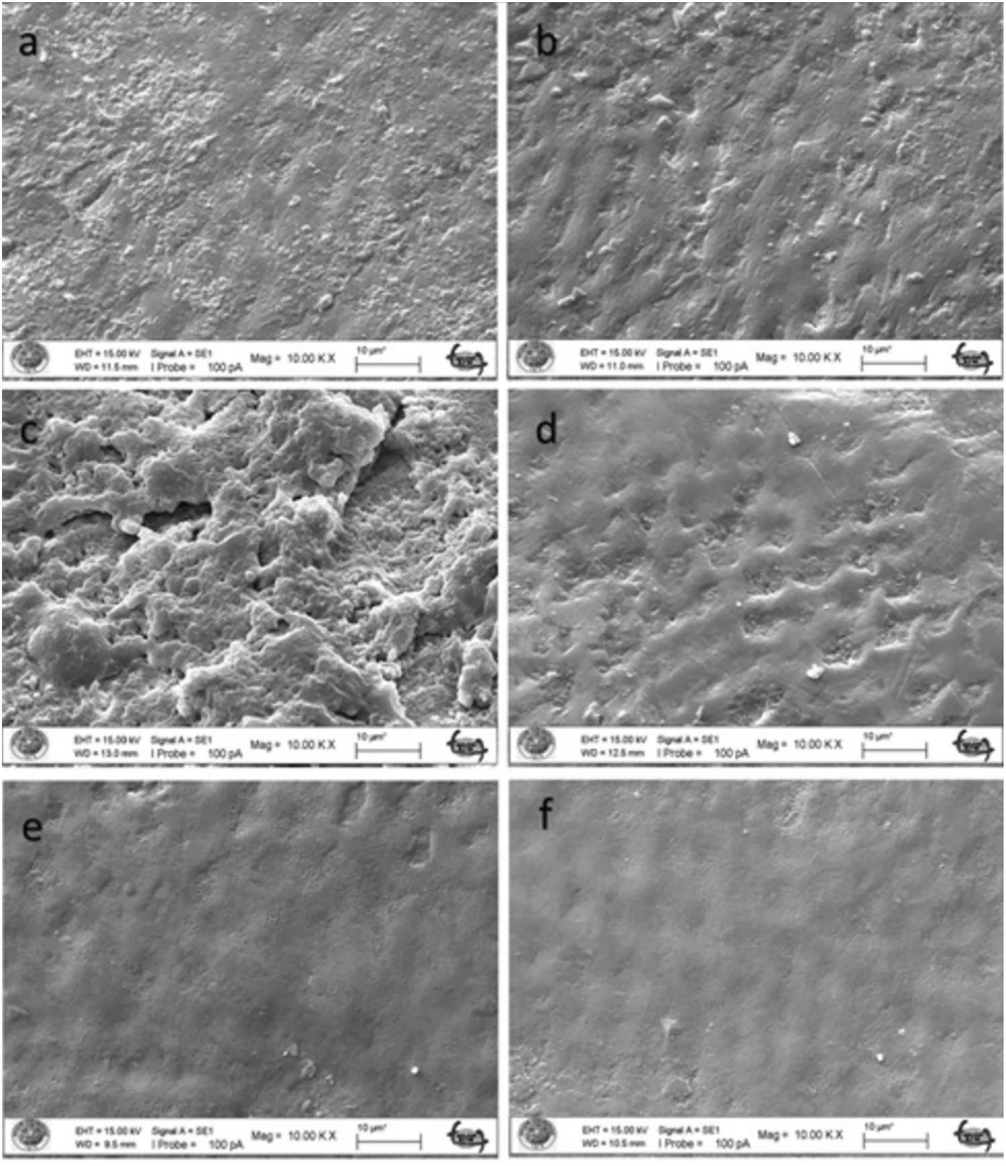
SEM image of enamel demineralised with acidic syrup. **a** FV applied enamel (× 10,000). **b** Enamel without FV applied (× 10,000). **c** SEM image of enamel with BAG varnish applied (× 5000). **d** Enamel without BAG applied (× 10,000). **e** SEM image of enamel with EPV applied (× 10,000). **f** Enamel without EPV applied (× 10,000)

The untreated enamel surface showed continuity and smoothness without any cracks or defects (Fig. 2a–b). When the enamel surface subjected to acid syrup erosion by fluoride varnish application was examined at 5000 × and 10,000 × magnification, a thin layer was covered with crystals deposited mainly around the pores (Figs. 3, 4a). When the acid syrup eroded surfaces were examined at 5000 × and 10,000 × magnification, a thin layer was covered with crystals deposited mainly around the pores in the fluoride varnish-treated groups. In the control groups, an irregular porous structure with depressions and protrusions was observed with surface demineralization of the enamel (Figs. 3, 4b). Micropores appeared with demineralization and the surface layer did not show the intact enamel surface structure (Figs. 3, 4b). The surface treated with BAG varnish was covered with a superficial compact amorphous layer containing a globular porous network and crystalline groups (Figs. 3, 4c). In the control groups, enamel cavitations, enamel prisms formed by surface demineralization and the formation of a honeycomb appearance were observed (Fig. 3d). Acid erosion caused a porous appearance and rough surfaces (Fig. 4d). No surface coverage findings were observed in the EPV varnish-treated groups (Figs. 3, 4e). In the control groups, a more demineralised appearance was observed compared with the enamel surface, in particular partial dissolution with the appearance of micropores (Figs. 3, 4f).

**Fig. 5:**
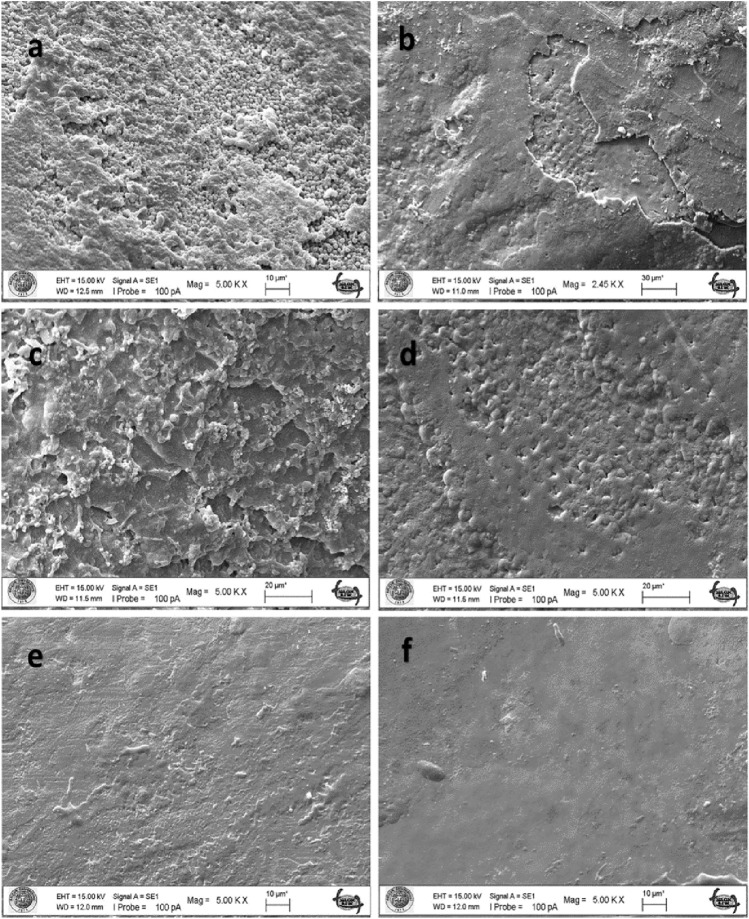
SEM image of enamel demineralised with acidic drink. **a** FV applied enamel (× 5000). **b** Enamel without FV applied (× 5000). **c** SEM image of enamel with BAG varnish applied (× 5000). **d** Enamel without BAG applied (× 5000). **e** SEM image of enamel with EPV applied (× 5000). **f** Enamel without EPV applied (× 5000)

**Fig. 6:**
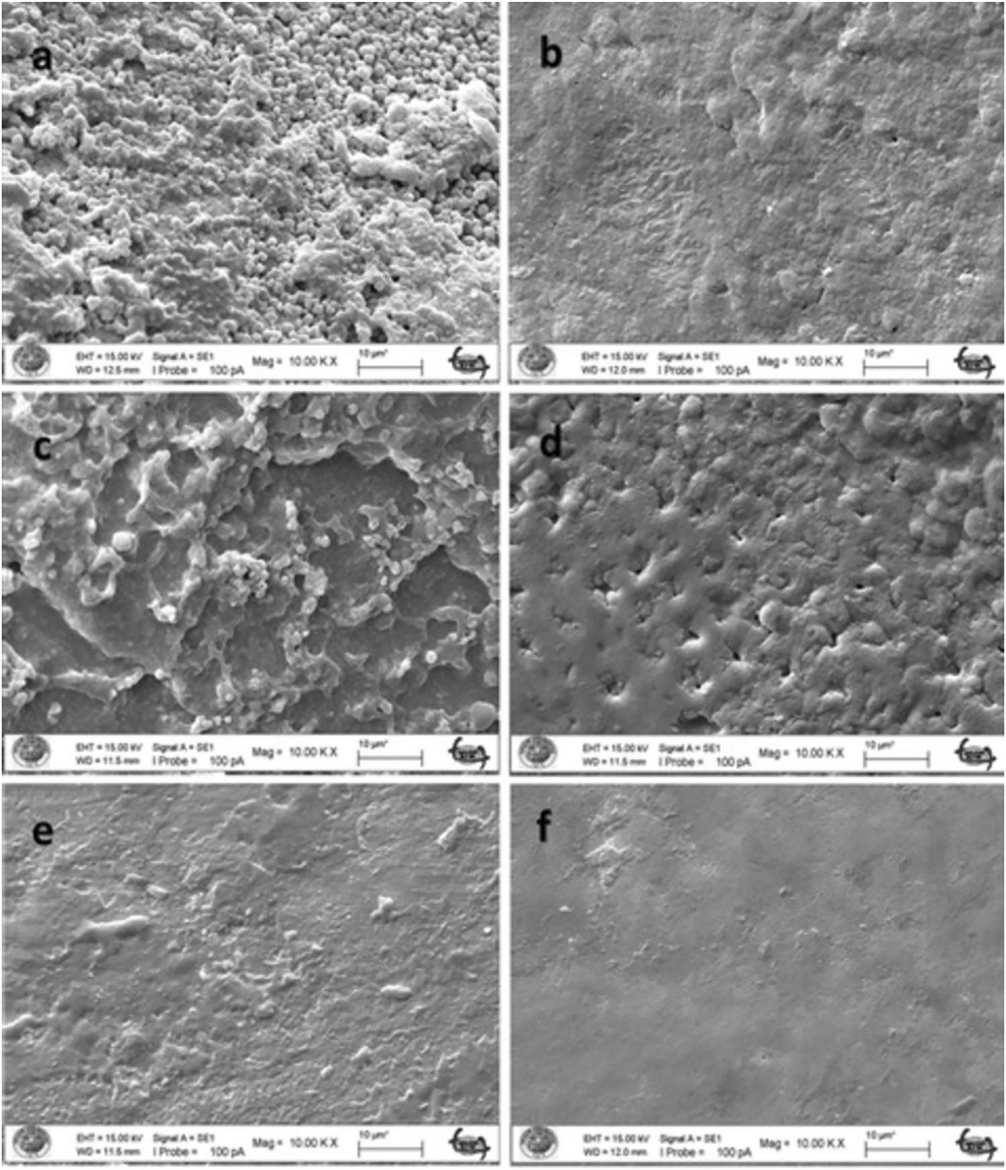
SEM image of enamel demineralised with acidic drink. **a** FV applied enamel (× 10,000). **b** Enamel without FV applied (× 10,000). **c** SEM image of enamel with BAG varnish applied (× 5000). **d** Enamel without BAG applied (× 10,000). **e** SEM image of enamel with EPV applied (× 10,000). **f** Enamel without EPV applied (× 10,000)

When the enamel surface eroded by the acidic drink was examined at 5000 × and 10,000 × magnification, it was covered with a layer of amorphous and spherical structures of different sizes in the fluoride varnish-treated groups (Figs. 5, 6a). The presence of rounded formations on the surface of the tooth specimen supports the possibility that these are CaF2-like spherical structures. In the control group, cracks and fissures were observed in the enamel (Fig. 5b). With the loss of inorganic structure on the enamel surface, an irregular porous structure with indentations was observed on the surface. It was observed that small cavitations were formed by the dissolution of crystals on the irregular enamel surface (Figs. 5, 6b). In the groups treated with BAG varnish, the surface was covered with a superficial compact amorphous layer containing a globular porous network and crystal groups (Figs. 5, 6c). Without the application of BAG varnish, acidic beverage erosion resulted in enamel cavitations, enamel prisms formed by surface demineralization and the formation of a honeycomb appearance (Fig. 5d). Acid erosion caused a porous appearance and rough surfaces (Fig. 6d). The characteristic honeycomb structure of demineralised enamel is clearly visible, with relatively deep but conical cauterized pits increasing in diameter up to about 10 microns (Fig. 6d).The YKZT varnish group was covered with a thin amorphous layer and scattered crystals deposited mainly around the pores (Figs. 5, 6e). In the control group, there was no evidence of surface demineralization of the enamel (Figs. 5, 6f).

### ATR-FTIR results

#### CO_3_^−2^ ve PO_4_^−3^ Peak spectrums

**Fig. 7:**
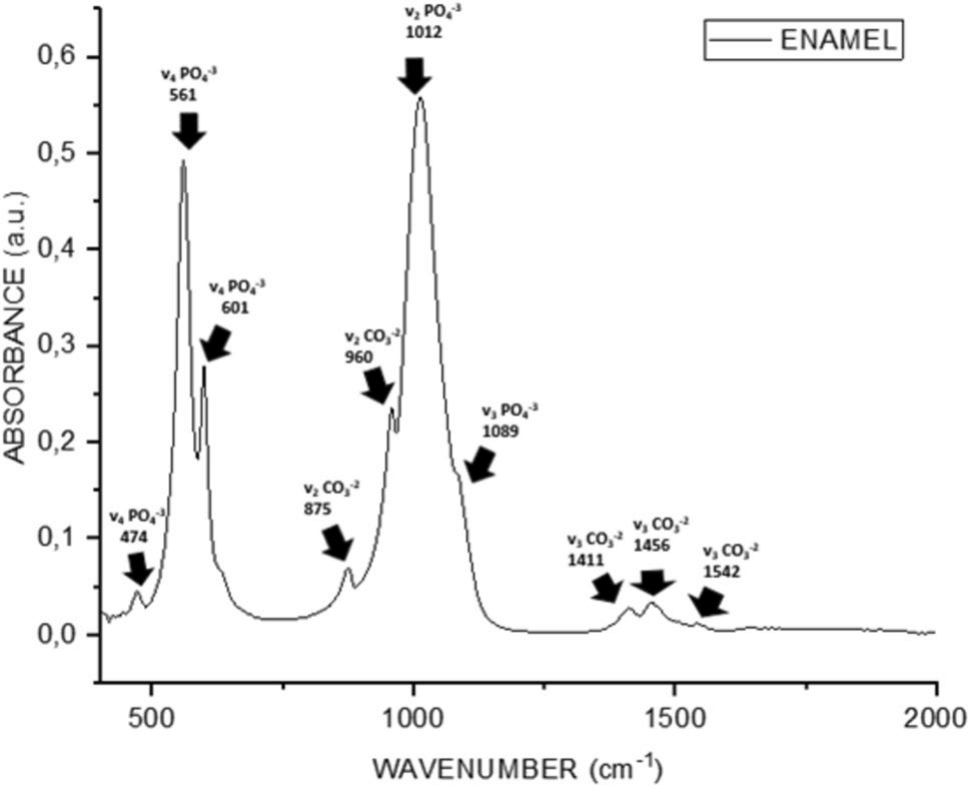
ATR-FTIR analysis of intact enamel surface

**Fig. 8:**
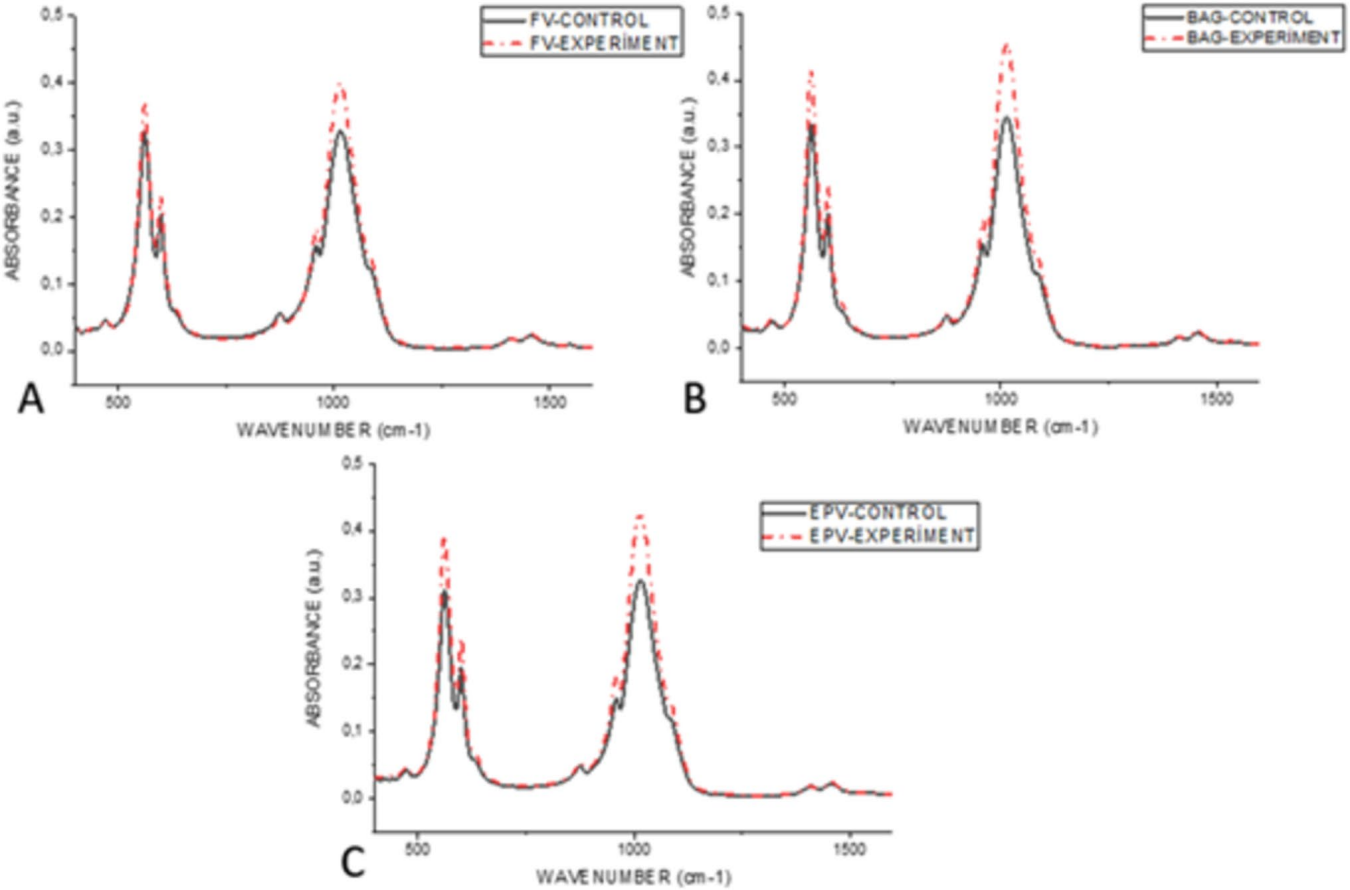
ATR-FTIR absorbance spectra of control and varnish applied areas of samples demineralised with acidic syrup. Red dashed line FV varnished black solid line control group (**A**). Red dashed line BAG varnished black straight line control group (**B**). Red dashed line Black solid line control group with EPV varnish (**C**)

**Fig. 9:**
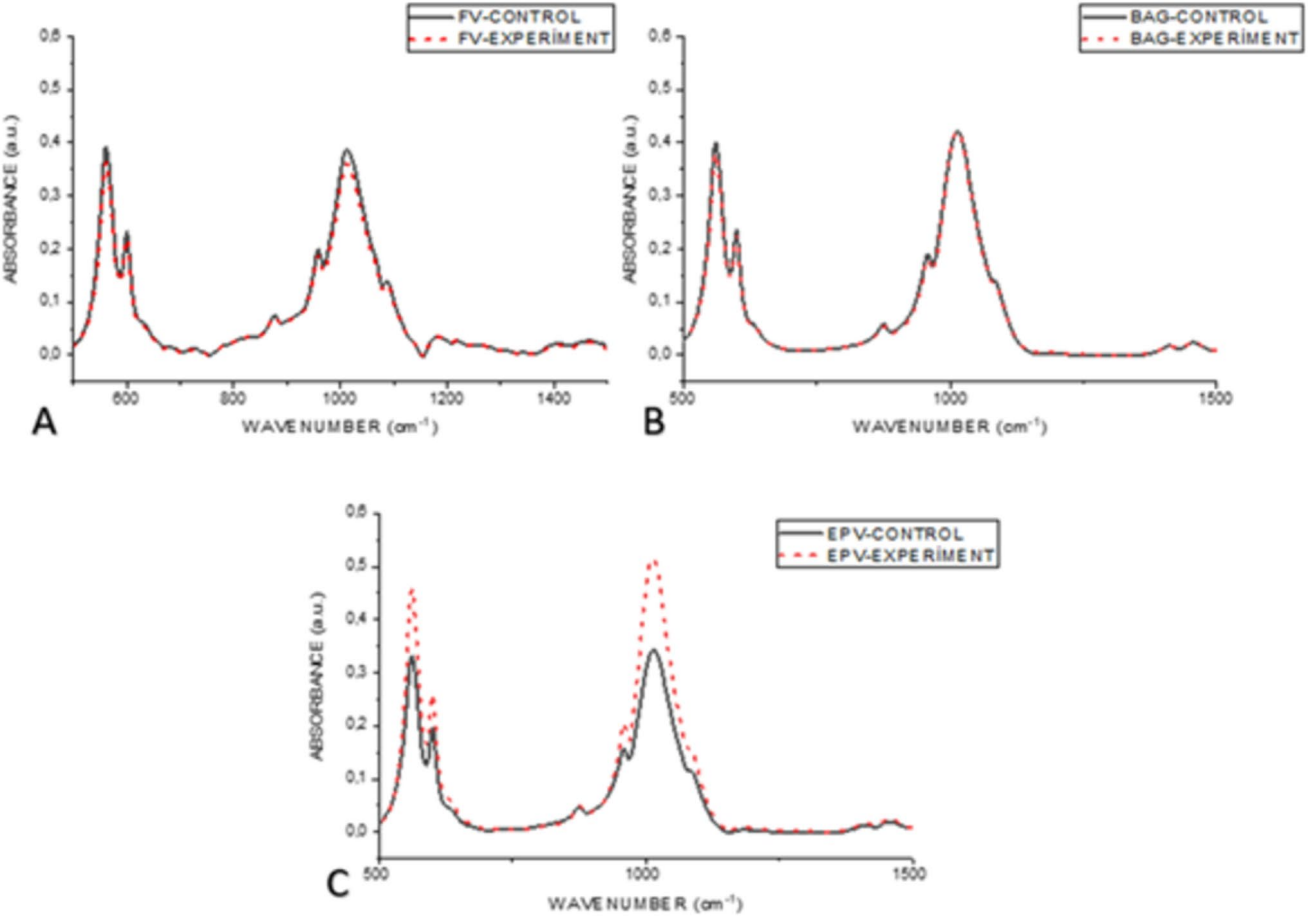
ATR-FTIR absorbance spectra of acidic drink demineralised samples from control and varnish applied areas. Red dashed line FV varnished Black solid line control group (**A**). Red dashed line BAG varnished black straight line control group (**B**). Red dashed line Black solid line control group with EPV varnish (**C**)

The mean ATR-FTIR spectrum fingerprint region of the enamel samples from the experimental and control groups exhibited phosphate and carbonate bands. These are 1542 (α-type v_3_ CO_3_^−2^), which also correspond to the spectra of the dental hydroxyapatite peak and band positions previously obtained in the literature [43]. Additionally, the following bands were identified: 1456 (α-type v_3_ CO_3_^−2^), 1411 (β-type v_3_ CO_3_^−2^), 1089 (v_3_ PO_4_^−3^), 1012 (v_3_PO_4_^−3^), 960 (v_1_ PO_4_^−3^), 875 (β-type v_2_ CO_3_^−2^), 601 (v_4_ PO_4_^−3^), and 561 (v_4_ PO_4_^−3^) (Figs 7, 8, 9).

A t test was employed to conduct within-group comparisons in accordance with the ATR-FTIR results. No statistically significant difference was observed when each group was compared with its control (p > 0.05). The mean values of the absorption intensities of the 875 peak, as analyzed by Fourier-Transform Infrared Spectroscopy (FTIR), of the control and varnish-applied areas of the groups demineralised with an acidic beverage, were found to be statistically significantly higher in the FV group than in the EPV group (p < 0.05).

#### Carbonate, v_1_, v_3_ phosphate, B-type v_1_ carbonate, v_1_ phosphate, type A v_3_ carbonate band areas

The band areas of v2 carbonate, v1 phosphate, B-type v1 carbonate, v1 phosphate, A-type v3 carbonate were employed to elucidate alterations in mineral properties within the enamel. Furthermore, alterations in the area under the phosphate and carbonate curves also serve to indicate the erosive effects of erosion [44]. The area calculations were performed using the OriginPro 8.5 software.

Following the basic correction of the field calculations from 1700 to 800 cm^−1^, the carbonate/phosphate ratio in the infrared spectrum was obtained by utilizing the ratio of the fields under the carbonate band v2 (850–890 cm^−1^) and the phosphate bands v_1_ and v_3_ (900–1200 cm^−1^) [45, 46]. The results were expressed as the mean and standard error of the mean for each group.

Carbonate CO_3_^−2^ v_2_ (850–890 cm^−1^), Phosphate v_1_ (850–890 cm^−1^), Carbonate CO_3_^−2^ v_2_ (850–890 cm^−1^) were analyzed by Fourier-Transform Infrared Spectroscopy (FTIR) of control and varnish-treated areas of groups demineralised with acidic syrup. There was no statistically significant difference (*p* < 0. 05) between the mean values of the integrated areas of v_3_ (900–1200 cm-1), Β**–**type carbonate (1050–1090 cm^−1^), v_1_ phosphate (930–980 cm-1), and α-type carbonate (1300–1650 cm^−1^) (Table3).

**Table 3:** Mean values of the integrated area of each chemical component analyzed using Fourier-Transform Infrared Spectroscopy (FTIR) of the control and varnish applied areas of the groups demineralised with acidic syrup

Integrated Band Areas		Carbonate v_2_ (850–890 cm^−1^)	Phosphate v_1_-v_3_ (900–1200 cm^−1^)	Β-Type Carbonate (1050–1090 cm^−1^)	v_1_ Phosphate (930–980 cm^−1^)	α Type Carbonate (1300–1650 cm^−1^)
*Acidic Syrup*		Mean SD	Mean SD	Mean SD	Mean SD	Mean SD
FV	Experiment	1,72 **0,17**	43,24 **6,63**	6,03 **1,12**	7,44 **1,03**	3,59 **0,65**
	Control	1,79 **0,31**	39,11 **7,57**	6,87 **0,99**	6,74 **1,43**	3,42 **0,56**
BAG	Experiment	1,63 **0,35**	45,69 **7,13**	6,84 **0,89**	7,70 **1,27**	3,09 **0,45**
	Control	1,56 **0,39**	36,58 **12,80**	5,52 **1,71**	6,45 **2,23**	2,80 **0,72**
EPV	Experiment	1,78 **0,49**	44,89 **12,53**	7,10 **1,51**	7,50 **2,47**	3,51 **0,79**
	Control	1,61 **0,43**	36,07 **10,50**	5,87 **1,43**	6,22 **1,92**	3,43 **0,67**

Carbonate CO_3_^−2^ v_2_ (850–890 cm^−1^) integrated field means of the groups demineralized with acidic drink were significantly different between BAG-C and FV, EPV, EPV-C-K groups and between FV and FV-C groups (*p* < 0.05). A significant difference was found between the mean values of phosphate v_1_, v_3_ (900–1200 cm^−1^) integrated fields between EPV-C and BAG and BAG-C groups (*p* < 0.05). No statistically significant difference was found between the mean values of phosphate v_1_ (930–980 cm-1), **α** type carbonate (1300–1650 cm-1), Β -type carbonate (1050–1090 cm^−1^) integrated fields (*p* > 0.05)(Table 4).

**Table 4:** Mean values of the integrated area of each chemical component analyzed using Fourier-Transform Infrared Spectroscopy (FTIR) of the control and varnish-treated areas of the acidic drink demineralised groups

Integrated Band Areas		Carbonate v_2_ (850–890 cm^−1^)	Phosphate v_1_-v_3_ (900–1200 cm^−1^)	Β-Type Carbonate (1050–1090 cm^−1^)	v_1_ Phosphate (930–980 cm^−1^)	α Type Carbonate (1300–1650 cm^−1^)
Acidic drink		Mean SD	Mean SD	Mean SD	Mean SD	Mean SD
FV	Experiment	1,49 **0,20**	42,15 **4,37**	6,47 **1,70**	7,36 **2,28**	1,97 **0,68**
	Control	1,90 **0,27**	44,37 **1,97**	6,87 **1,20**	7,82 **1,89**	2,18 **0,71**
BAG	Experiment	1,80 **0,20**	47,99 **9,13**	6,77 **2,33**	7,42 **1,83**	2,52 **0,61**
	Control	2,05 **0,21**	47,40 **5,51**	6,95 **1,35**	7,90 **2,02**	2,64 **0,09**
EPV	Experiment	1,64 **0,10**	45,88 **1,94**	8,08 **0,78**	8,56 **1,00**	2,39 **0,52**
	Control	1,58 **0,21**	37,21 **4,90**	5,50 **1,70**	5,99 **2,17**	2,33 **0,47**

#### Carbonate/phosphate ratios

**Fig. 10:**
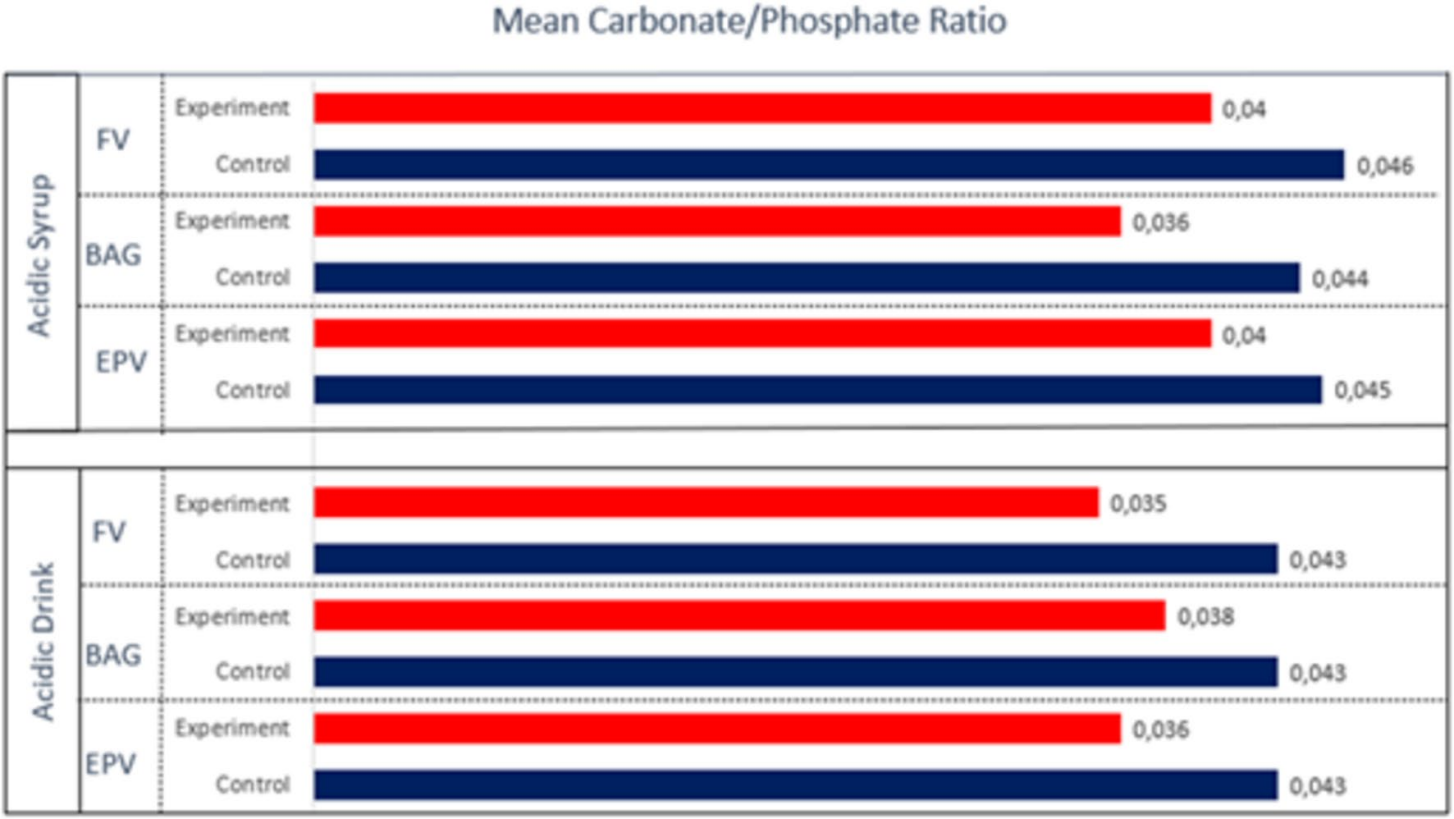
Mean values of carbonate/phosphate ratios analyzed using Fourier-Transform Infrared Spectroscopy (FTIR) for the control and varnish-treated areas of the demineralised groups All

The average carbonate/phosphate ratios in the groups obtained from the ATR-FTIR spectra are shown in Fig. 10. Among the carbonate/phosphate ratios analyzed by Fourier-Transform Infrared Spectroscopy (FTIR), the carbonate/phosphate ratios of the control groups were higher than those of the varnish groups, but no statistically significant difference was found in the comparisons (*p* > 0.05).

### Microhardness results

**Fig. 11:**
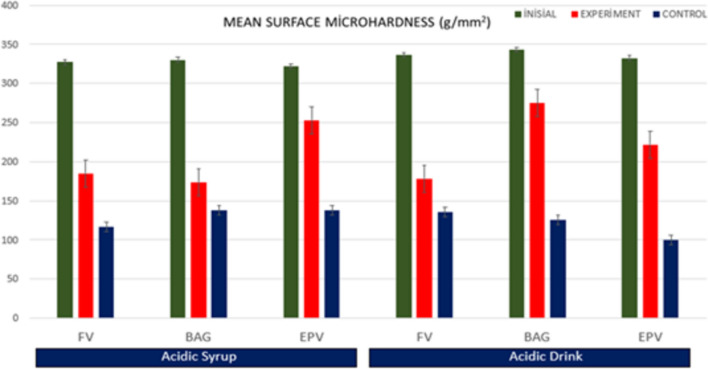
Distribution of surface VSN values obtained at baseline, after demineralization and after pH cycling in experimental and control groups

The mean surface microhardness (VSN) values of the initial, demineralization and pH cycling varnish treated and control groups obtained from a total of 6 groups in the study are given in Fig .11. It was found that the initial VSN (g/mm2) values obtained in all groups were significantly higher and statistically different from the VSN values of the control and varnish groups (*p* < 0.05).

All acid syrup demineralized varnish groups showed higher microhardness values than the control group, but a statistically significant difference was found between FV and FV-C (*p* = 0.23) and EPV and EPV-C (*p* = 0.00). A statistically significant difference was found between FV and BAG and EPV (*p* = 0.25).

All groups treated with acidic drink demineralized varnish showed higher microhardness values than the control group, but a statistically significant difference was found between BAG and BAG-C and EPV and EPV-C groups (*p* < 0.05). When compared between groups, a statistically significant difference was found between the mean microhardness of the FV and EPV groups (*p* = 0.034). Fig. 11.

## Discussion

In this study, fluoride varnish, varnish containing bioactive glass, varnish containing sodium tri-metaphosphate-treated eggshell and membrane powder were applied to enamel surfaces and exposed to acidic syrup and acidic beverage erosion cycles, and the erosion protection of these varnishes was evaluated in vitro. The null hypothesis of this in vitro study ‘There is no difference between the effects of fluoride varnish, bioactive glass varnish, sodium tri-metaphosphate-treated varnishes and varnishes containing eggshell and membrane powder on protection against erosion caused by non-alcoholic acidic beverage and acidic drug’ was partially rejected. In the comparison between the groups, there was a difference between the FV (Polimo varnish) and EPV (BioViera varnish) groups in the 875 cm^−1^ v_2_ CO_3_^−2^ peak spectrum and in the microhardness values. There were no differences between the groups for other parameters. In general, BAG and EPV varnishes showed similar results to FV varnishes.

There are conflicting results in the literature, ranging from no or limited protection of topical fluoride against tooth erosion [47–49] to almost complete protection [50]. This may be due to differences in study design, particularly with regard to the type of dental substrate and the pH and concentration of the different fluoride substances used [51]. The SEM results of our study confirm the protective effect of fluoride against demineralization by acidic drinks as seen in previous studies [52, 53]. According to the SEM results, it was observed that the coating formed on the surface of the FV and EPV groups demineralized with acidic syrup was thinner than that of the acidic beverage groups and the surface was similar to natural enamel. In the BAG groups, a clear surface coating was found after both acidic syrup and acidic drink demineralization.

Sodium tri-metaphosphate (STMP) is a condensed inorganic phosphate. It can bind strongly to phosphate sites on the enamel surface and remain adsorbed longer than other phosphates. This leads to the formation of a protective layer on the enamel surface, which limits the diffusion of acid ions during the development of dental caries [32]. This protective barrier limits the diffusion of calcium and fluoride ions from the enamel [32]. In situ models have shown that the addition of STMP to a low fluoride product produces remineralization effects similar to a 1100 ppm fluoride formulation [54].

In our study, varnish (Bio Viera varnish) containing eggshell and membrane powder treated with fluoride-free STMP was used. According to the SEM findings of the study, STMP-treated eggshell and membrane powder-containing varnish (Bio Viera varnish) showed a thin amorphous layer on the surface against acidic beverage erosion and scattered crystals deposited mainly around the pores, but no protective layer was observed in the acidic syrup group, but no signs of erosive demineralization were observed.

Nanohydroxyapatite is commonly incorporated into toothpaste to promote enamel remineralization and treat dentin hypersensitivity. Onwubu et al. report on the remineralization and acid-resistant properties of nHAp produced from eggshell by mechanochemistry. The results show that nHAp has good buffering properties against acids [55]. In our study, the SEM image of the surface treated with EPV varnish also showed that the effect on enamel surface integrity in the presence of nHAp was minimal, suggesting that it is a form of enamel remineralization. This may be due to the eggshell and the buffering capacity of STMP and nHAp within it, preventing enamel dissolution.

FTIR is based on molecular vibrations experienced by different groups of atoms of the mineral structure (e.g., PO_4_^3−^) and functional groups of the organic components of the tooth (e.g., collagen amides). The area or height of the bands is used as a measure for quantitative analysis of the major components present in dental samples [56]. In our study, the peaks and areas of these bands were analyzed.

The mineral matrix of enamel consists largely of crystals of carbonate hydroxyapatite [57] and the components absorbed in the infrared are hydroxyl (OH), carbonate (CO_2_^−3^) and phosphate radical (PO_3_^−4^) [58]. In contrast to the pure hydroxyapatite crystal with the formula Ca_10_ (PO_4_)^6^ (OH)_2_, enamel contains large ionic substitutions. Of these, the F- ion can replace OH by CO_3_^2−^, PO_4_^3−^ or OH^−^, and Na^+^ can replace Ca^2+^. Most of these ion substitutions significantly increase the solubility of the tooth mineral, which is represented by the simplified formula [59]. Within the nano-sized HA lattice structure, anionic CO_3_^−2^ ions are replaced by OH^−^ groups to form α-type carbonate or tetrahedral PO_4_^−3^ to form β-type carbonate [59, 60].

In enamel, carbonate, CO_3_^−2^, can replace PO_4_^3−^ or OH^−^ ions (α-type carbonate) at two anionic sites in the apatite lattice (β-type carbonate). According to previous studies, it shows a very weak absorption band at about 1540 cm^−1^ and weak bands at 1453 cm^−1^ (contribution from the A and B regions) and around 1420 cm^−1^ (B region) in the carbonate stretching mode, with higher wave numbers being more intense. In the deformation mode, there is a net carbonate absorption band at 872 cm^−1^ [58, 61, 62]. Our study showed similar enamel spectra and band positions to previous studies.

When the acid syrup demineralized groups were compared with the control, an increase in the absorbance intensities of the CO_3_^−2^ and PO_4_^−3^ peaks was observed, but this increase was not statistically significant. In the acid demineralised groups, these apparent peaks were observed only in the EPV group as shown in Fig. 11. This may be due to the higher pH of the acidic syrup and natural remineralization may occur in the EPV group demineralised with acidic drink.

A significant decrease in the intensity of the ν_1_ PO_4_^−3^ band at 960 cm^−1^ indicates a change in the apatite structure during erosion. The relative carbonate content can be determined by vibrational spectroscopy (IR & Raman) of apatites. In our study, lower values were found in the ν_1_ PO_4_^−3^ band at 960 cm^−1^ in acid syrup erosion in all groups in the control groups eroded without varnish application, but there was no statistical difference. In the acidic beverage group, lower values were observed only in the fluoride varnish control group, while the values in the other groups were close to each other. The lower pH of the acidic syrup may have caused the erosion findings to be more pronounced.

The role of calcium and phosphate ions is related to maintaining tooth health and promoting the formation of various remineralisations in the tooth structure. The band assigned to the ν_4_ PO_4_^3^ vibration has been shown to be useful in analyzing the crystallinity of apatite, while the ν_3_ and ν_1_ modes of phosphate (959–1230 cm^−1^) have emerged as the best markers for determining the crystallinity of apatite [63]. The carbonate/phosphate ratio was lower in the lacquer groups than in the controls in all groups. This indicates that phosphate groups, which are a sign of remineralization, are more intense in the varnish groups.

Decreased hardness of enamel surfaces is an early sign of erosion [64]. Therefore, the abrasive potential of acidic substances on enamel surfaces can be measured by microhardness measurements, which are widely used to study enamel erosion [65]. In our study, the FV group showed similar microhardness values to the BAG varnish group and different values to the EPV group against acid syrup erosion. Against acidic beverage erosion, the BAG group showed similar microhardness values to FV and different from the EPV group. Against both erosions, the EPV groups showed the microhardness values closest to the initial values. In the SEM findings, the EPV groups did not show the protective layer seen on the surface of the other groups and an image close to the natural enamel image was obtained. The fact that the microhardness values obtained were also close to natural enamel, as mentioned above, we can say that the presence of STMP and nHAp in the EPV group creates a form of enamel remineralization without forming an additional layer on the surface.

## Conclusion

Erosion is a preventable disease. Varnishes are a good alternative to increase the resistance of teeth to acid. Patients should be advised to take precautions against erosion, taking into account the pH of syrups used for long-term asthma and allergy treatment and frequently consumed acidic beverages. As the fluorinated BAG and EPV varnishes used in our study showed similar results to FV, these bioactive products can be considered as an alternative for clinical use. However, the results of our study showed that the use of these varnishes alone will not be sufficient to prevent erosion unless the etiology is eliminated. In clinical practice, renewal of varnishes at regular intervals will increase their protective effect against acid erosion.
